# Energy metabolism modulates the regulatory impact of activators on gene expression

**DOI:** 10.1242/dev.201986

**Published:** 2024-01-02

**Authors:** Sha Qiao, Sebastian Bernasek, Kevin D. Gallagher, Jessica O'Connell, Shigehiro Yamada, Neda Bagheri, Luis A. N. Amaral, Richard W. Carthew

**Affiliations:** ^1^ Department of Molecular Biosciences, Northwestern University, Evanston, IL 60208, USA; ^2^ Department of Chemical and Biological Engineering, Northwestern University, Evanston, IL 60208, USA; ^3^ NSF-Simons Center for Quantitative Biology, Northwestern University, Evanston, IL 60208, USA; ^4^ Northwestern Institute on Complex Systems, Northwestern University, Evanston, IL 60208, USA; ^5^ Department of Physics and Astronomy, Northwestern University, Evanston, IL 60208, USA

**Keywords:** *Drosophila*, Gene regulation, Metabolism

## Abstract

Gene expression is a regulated process fueled by ATP consumption. Therefore, regulation must be coupled to constraints imposed by the level of energy metabolism. Here, we explore this relationship both theoretically and experimentally. A stylized mathematical model predicts that activators of gene expression have variable impact depending on metabolic rate. Activators become less essential when metabolic rate is reduced and more essential when metabolic rate is enhanced. We find that, in the *Drosophila* eye, expression dynamics of the *yan* gene are less affected by loss of EGFR-mediated activation when metabolism is reduced, and the opposite effect is seen when metabolism is enhanced. The effects are also seen at the level of pattern regularity in the adult eye, where loss of EGFR-mediated activation is mitigated by lower metabolism. We propose that gene activation is tuned by energy metabolism to allow for faithful expression dynamics in the face of variable metabolic conditions.

## Teaser

**Highlighted Article:** Mathematical modeling and experimental manipulation of the expression dynamics of *yan* in *Drosophila* eye demonstrate that cellular metabolic rate affects gene activation, with a reduced impact of activators under conditions of reduced metabolism.

## INTRODUCTION

The development of an organism occurs over a period of time that is distinct for the species to which the organism belongs (Ebisuya and Briscoe, 2018). Because development is coupled to the activities of gene regulatory networks (GRNs) that operate within cells, the dynamical properties of these GRNs are thought to influence the timing of development. For example, the turnover rates of proteins operating within a human neurodevelopmental GRN are slower than for those proteins operating in the homologous mouse GRN, and this difference is thought to partially explain the large difference in developmental tempo between the two species (Matsuda et al., 2020; Rayon et al., 2020).

The pace of development is also dependent upon extrinsic factors such as cellular metabolism (Iwata et al., 2023). Gene expression requires the continual synthesis of key metabolites and ATP, the primary source of chemical energy. The energy budget of a cell is composed of many competing processes that expend energy by consuming ATP. For example, during embryogenesis, gene expression and cell division account for a small fraction of the energy expended, suggesting that the energy budget is devoted to many biochemical processes (Rodenfels et al., 2019; Song et al., 2019). Energy expenditure is balanced with the generation of ATP, which depends on metabolic processing of nutrients. If an organism's nutrient uptake varies for whatever reason, then energy expenditure must accordingly adjust to prevent exhaustion of ATP stores (Locasale and Cantley, 2011). This principle has been demonstrated in *Drosophila* larvae as they grow and develop. Targeted ablation of insulin-like peptide (dILP) expression causes larval cells to reduce their uptake of circulating sugars by ∼40% (Zhang et al., 2009). There is a corresponding 30% decrease in energy expenditure by the body, and, as a result, the animals develop more slowly and grow into slightly smaller adults (Zhang et al., 2009). Developmental gene expression dynamics are correspondingly slower (Cassidy et al., 2019).

Although ATP content remains fairly constant in cells facing limited respiration, the fluxes of ATP synthesis and turnover are affected, manifesting in altered ratios of ATP to ADP and free phosphate (Brown, 1992). Anabolic processes are highly dependent on the ATP/ADP ratio (Atkinson, 1977). Because cells adjust their gene expression dynamics to variable energy budgets, this could theoretically occur in an unregulated manner simply based on dependence on the ATP/ADP ratio. However, there might also exist regulatory mechanisms within GRNs that provide coupling of expression dynamics to energy budgets. One such mechanism has been described for developmental GRNs in *Drosophila* (Cassidy et al., 2019). Using ablation of dILP-secreting cells to reduce energy expenditure in larval cells, it was found that repressors of gene expression became dispensable for their regulatory functions on target genes. This phenomenon was so pervasive that when energy metabolism was reduced, the entire family of microRNA repressors could be eliminated with minimal effect on *Drosophila* development (Cassidy et al., 2019). In contrast, under normal metabolic conditions, *Drosophila* microRNAs are essential for life (Pressman et al., 2012).

A stylized mathematical model was developed to explain this phenomenon, predicated on the observed expression dynamics of many genes involved in development (Cassidy et al., 2019). Developmental genes are often expressed in a succession of pulses, acting to successively restrict cell potential (Orkin and Zon, 2008; Jaeger et al., 2012; Cepko, 2014; Pollington et al., 2023). If genes require repressors to relax the pulse back to an off state, then when energy metabolism is reduced, the kinetics of pulse relaxation are naturally slowed, mitigating the need for a full complement of repressors as repressor molecules have more time to completely act on their targets (Cassidy et al., 2019). The model, despite its simplicity, captures the processes at play, explaining why many genes are individually regulated by multiple repressors. When energy metabolism is elevated, auxiliary repressors would provide supplementary aid in relaxing expression pulses, thus giving cells greater robustness to fluctuations in nutrient availability.

The broad implication of the findings by Cassidy et al. (2019) is that successful development is conditional upon the ability of GRNs to faithfully couple gene expression dynamics to variable levels of energy metabolism. However, the study only probed that relationship in the context of repressors operating at or below normal levels of energy metabolism. In this study, we explore two complementary possibilities. First, that activators of gene expression likewise become dispensable in developmental GRNs when energy metabolism is reduced, and second, that increasing metabolism beyond normal levels results in a greater need for auxiliary regulation. Both theoretical modeling and experiments support these two hypotheses. Thus, auxiliary gene activators also provide greater robustness to fluctuations in nutrient availability.

## RESULTS

### A dynamical model describes the relationship between gene activation and energy metabolism

**Fig. 1.**
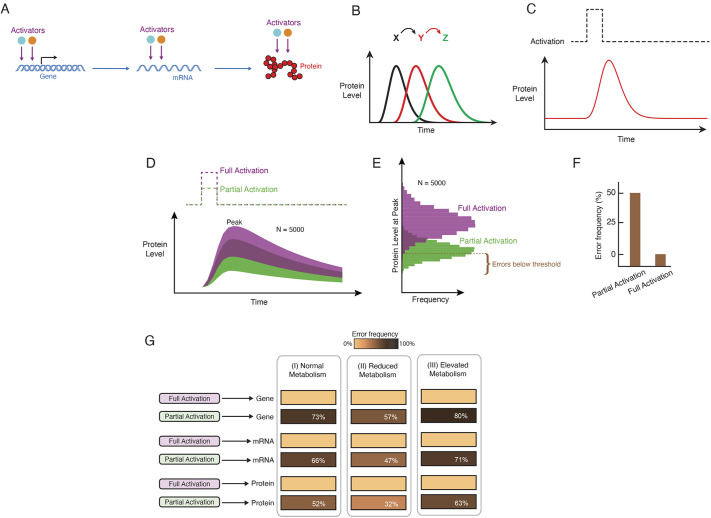
**Mathematical modeling of gene activation and the effects of energy metabolism.** (A) Schematic of generic regulation of gene expression. Multiple activators may act in concert to regulate expression at several levels. (B) Cascade of successive pulses in expression of three genes, products of which regulate one another as indicated at top. This program of gene expression occurs as a cell passes through a series of developmental states. The model focuses on transient expression of a single gene within the cascade. (C) Schematic of protein output from a single gene over time with a transient step function in gene activation followed by repression, turnover, and dilution to relax expression to a baseline state. (D) Model simulations showing protein output over time in response to a transient input signal. Shown are 5000 simulated trajectories, which merge into a continuous band of trajectories. Green and purple denote simulations with 50% (partial) and 100% (full) activation of gene expression, respectively. Dark purple denotes overlap of trajectories. (E) Frequency distribution of peak-level protein output from all simulations. A threshold is set (dashed line) where 99% of simulations with full activation are greater in peak output. (F) With partial activation, fewer simulations cross the threshold. Each failure to cross the threshold is an error. (G) Error frequency is greater with impaired activation at the transcriptional, RNA and translational steps of gene expression. However, impaired activation imparts fewer errors when ATP-dependent parameter values are reduced by 50%, and impaired activation imparts more errors when ATP-dependent parameter values are increased by 50%, regardless of how activators act on gene expression.

We modified the mathematical model previously developed by Cassidy et al. (2019) to test the hypothesis that activators of gene expression become dispensable when energy metabolism is reduced. Conceptually, the modified model describes a simplified pathway of gene expression that represents the expression of a single gene (Fig. 1A). If the gene encodes an activator of other genes in a cascade, then simple GRN circuits can generate pulsatile dynamics (Fig. 1B). Indeed, such circuits are commonly found in developmental GRNs (Orkin and Zon, 2008; Jaeger et al., 2012; Pollington et al., 2023).

In our control theoretic model, activators transiently stimulate expression of a gene, with the associated protein expression as the output (Fig. 1C). Each step of gene expression is potentially mediated by one or more activators acting in parallel at the levels of mRNA transcription, mRNA processing/stability, and protein translation (Fig. 1A). Combined, these activators help determine the size of the output's pulse amplitude. mRNA and protein turnover lead to a relaxation in output back to a baseline level (Fig. 1C). Because gene expression is noisy (Arias and Hayward, 2006), we used a stochastic simulation approach to infer the entire distribution of possible dynamic trajectories in protein output. There was a broad distribution of pulsatile trajectories from 5000 such stochastic simulations (Fig. 1D, purple traces).

We then compared the output dynamics when gene activation was reduced by 50% (i.e. one or more activators were absent) (Fig. 1D, green traces). The two distributions partly overlapped but a large fraction of simulations with partial activation gave diminished protein output. This effect was consistently observed over a broad range of model parameter values, with 1000 parameter sets tested (Fig. S1A).

Because each pulse must trigger subsequent events in the GRN cascade, propagation of the cascade is contingent upon sufficient peak expression of each gene. Activators are crucially important for the peak level (amplitude) of each pulse; peak output level was generally reduced when gene activation was reduced (Fig. 1E). We defined a minimum amplitude that the output level must reach before a subsequent event is triggered. This threshold was defined such that exactly 99% of simulations with full activation achieved sufficient pulse amplitude to trigger a subsequent pulse (Fig. 1E). The remaining 1% of trajectories that failed to reach the threshold level were denoted ‘errors’. Errors became much more frequent with partial gene activation (Fig. 1E,F). This property was observed over a broad range of model parameter values (Fig. S1B,C), and regardless of whether activators function in transcription, RNA processing, or protein translation (Fig. 1G).

**Fig. 2.**
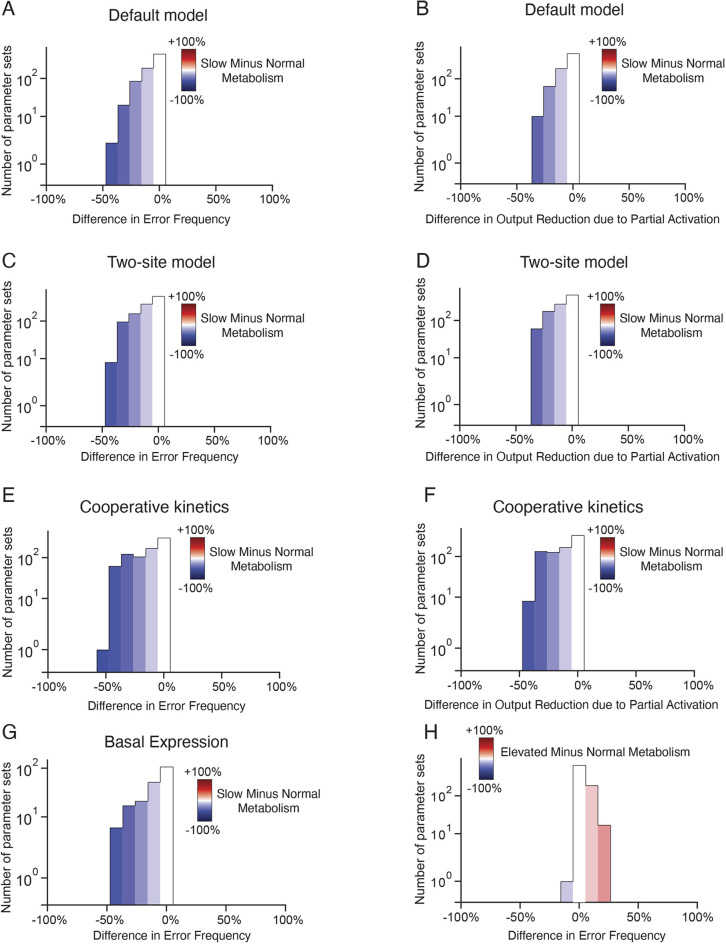
**Model predictions are robust to model feature variation.** Each of the seven model parameters was varied by one order of magnitude centered around the default value as defined in the Materials and Methods section. For each parameter set, 1000 parameter sets were generated, and 5000 simulations with full and partial activation were performed. (A-G) Systematic modification of model conditions showing the difference between reduced (slow) versus normal metabolism for all parameter sets. For A,C,E,G, protein output at the peak of expression was compared between full and partial activation. Error frequency from partial activation was estimated using a threshold in peak expression. Shown are the distributions of the difference in error frequency between reduced and normal metabolism for all parameter sets. For B,D,F, protein output was calculated over the entire time course of gene expression, and the frequency with which output reduction occurred with partial activation was estimated for all parameter sets. Shown are the distributions of the difference in output reduction between reduced and normal metabolism for all parameter sets. (A,B) The default model. (C,D) Model in which an upper bound of two is placed on the number of alleles transcribing the gene. (E,F) Model in which cooperative transcription kinetics are considered. (G) Model where a non-zero basal stimulus is applied. (H) In the default model, error frequency from partial activation was estimated using a threshold in peak expression. Shown are the distributions of the difference in error frequency between elevated and normal metabolism for all parameter sets.

**Fig. 3.**
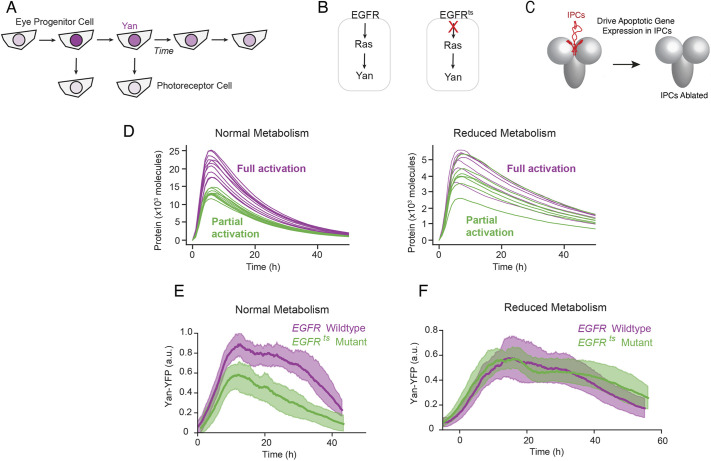
**EGFR activation of Yan expression is dispensable when metabolism is slowed.** (A) Schematic of Yan expression dynamics in eye progenitor and photoreceptor cells. Depth of purple color indicates level of expression. (B) Yan expression is positively dependent on EGFR in eye progenitor cells. (C) The 14 IPC cells in the larval brain are killed by specific expression of the pro-apoptotic protein Rpr. (D) Simulated protein output under the control of an auxiliary transcriptional activator (purple), and when the activator is removed (green). All simulations (purple and green) are also under control of a constitutive activator. Shown are ten randomly chosen samples from a total population of 5000 trajectories for each condition. Left: Simulations performed with normal ATP-dependent reaction rates. Right: Simulations performed following a 50% reduction in the rate of ATP-dependent reactions. (E,F) Yan-YFP expression dynamics in eye progenitor cells that are wild-type *EGFR* (*EGFR^tsla^/EGFR^+^*) or mutant *EGFR* (*EGFR^tsla^/EGFR^f24^*) raised at the semi-permissive temperature. Time 0 marks the time at which Yan expression begins. Solid lines are moving averages. Shaded regions denote 95% confidence intervals. Each line average is calculated from a composite of measurements of between 4448 and 5406 cells. (E) Yan-YFP dynamics under normal metabolic conditions in with either *UAS-Rpr* or *dILP2-Gal4* in the genetic background. (F) Yan-YFP dynamics when the IPCs have been ablated (*dILP2-Gal4/UAS-Rpr*). a.u., arbitrary units.

We next investigated whether gene activation is less essential for peak protein output when energy metabolism is reduced. We reduced the rate parameters of each ATP-utilizing reaction by 50% to reflect conditions of reduced energy metabolism and compared simulation outcomes with full gene activation versus partial gene activation. The error frequency induced by partial gene activation was significantly diminished when ATP-dependent rate parameters were lowered (Fig. 1G). This effect on activator loss occurred when activators function in transcription, RNA processing, or protein translation (Fig. 1G), and it persisted across a wide range of parameter values (Fig. 2A). The same effect was also evident when comparing cumulative output protein expression rather than the instantaneous peak levels reached by each expression pulse (Fig. 2B). Furthermore, the effect persisted when (1) an upper bound of two alleles was placed on the gene's transcription output (Fig. 2C,D), (2) cooperative transcription kinetics were taken into account (Fig. 2E,F), and (3) there was a non-zero basal level of gene expression (Fig. 2G).

We then examined whether elevating energy metabolism above normal exacerbates error frequency. To simulate the effect of elevating energy metabolism above normal, we increased the rate parameters of each ATP-dependent reaction by 50% and found that error frequency was enhanced when gene activation was impaired (Fig. 1G). This effect on activator loss occurred when activators function in transcription, RNA processing, or protein translation (Fig. 1G), and it was observed across a wide range of parameter values (Fig. 2H). Combined, all of our simulations predict that protein output of gene expression is differentially sensitive to changes in gene activation when energy metabolism is varied.

### Experimental validation of the dynamical model

We experimentally tested the model's key prediction by measuring the expression dynamics of the Yan (Aop) protein in the *Drosophila* larval eye. Yan exhibits pulsatile expression in progenitor cells of the eye, and its expression is activated by a wave of transient signaling through the Epidermal Growth Factor Receptor (EGFR) (Boisclair Lachance et al., 2014; Peláez et al., 2015). Eye progenitor cells rapidly upregulate Yan protein abundance to a peak; the protein then decays back to initial levels over the course of 40 h (Fig. 3A). Loss of EGFR results in a twofold reduction in peak Yan expression in progenitor cells (Peláez et al., 2015). As progenitor cells achieve peak levels of Yan, some of them are induced to transition to photoreceptor fates (Fig. 3A) (Peláez et al., 2015; Bernasek et al., 2023). Yan protein does not promote the transition but actually inhibits the transition (Voas and Rebay, 2004). Interestingly, a second wave of transient EGFR signaling induces these fate transitions by triggering the rapid downregulation of Yan protein levels (Fig. 3A) (Voas and Rebay, 2004). Thus, EGFR acts as an activator of Yan in progenitor cells and a repressor of Yan in photoreceptor cells.

To measure Yan protein precisely in the progenitor cells, we used a *Drosophila* strain in which the Yan protein is tagged with YFP and is still fully functional (Boisclair Lachance et al., 2014; Peláez et al., 2015). Confocal microscopy of the eye disks was coupled with a computational pipeline for segmentation and analysis, yielding a composite picture of Yan dynamics sampled from thousands of cells per condition (Peláez et al., 2015; Bernasek et al., 2023).

Previous observations show that the pulse of Yan expression in progenitor cells is activated by EGFR (Peláez et al., 2015). When we transiently raised a temperature-sensitive (ts) *EGFR* mutant at a semi-permissive temperature of 26.5°C, peak output of Yan protein was reduced by ∼35%, whereas Yan output was unaffected by the *EGFR^ts^* mutant at a permissive temperature of 18°C (Figs S2 and S3).

We genetically ablated the insulin-producing cells (IPCs) of the larval brain (Fig. 3C) by driving expression of the pro-apoptotic protein Reaper (Rpr) in IPCs using a *dILP2-Gal4* driver. dILP2 (Ilp2) is a major insulin-like peptide produced by the IPCs (Rulifson et al., 2002). Ablation of the IPCs using this system causes a 40% reduction in cell uptake of circulating sugars, a decrease in mitochondrial oxidative phosphorylation, and a 70% slowdown in overall fly development (Cassidy et al., 2019; Rulifson et al., 2002). We ablated the IPCs and observed that, under these conditions, loss of EGFR had little to no effect on Yan expression (Fig. 3F), in contrast to the effect seen under conditions of normal metabolism (Fig. 3E). This behavior resembled the simulated modeling dynamics under conditions of normal and reduced energy metabolism (Fig. 3D). Model simulations predicted that the amplitude of protein output will be less sensitive to impaired gene activation if energy metabolism is reduced.

Recently, it was found that EGFR acts in a set of IPC-connecting neurons (ICNs) (Meschi et al., 2019). When EGFR is activated in ICNs, they stimulate secretion of dILP2 from the IPCs. This raised the possibility that the dependence of Yan on EGFR might simply be due to an indirect effect of EGFR acting in the ICNs to modulate dILP2. Consequently, if activation of Yan expression is mediated by dILP2, then ablation of the IPCs would naturally suppress this dependence. To test the possibility, we measured dILP2 protein levels in IPCs from the *EGFR^ts^* mutant raised at 26.5°C. There was no significant difference in dILP2 accumulation in mutant IPCs compared with wild-type control (Fig. S4). If dILP2 secretion had been inhibited in the mutant, we would have observed elevated dILP2 accumulation in IPCs, as occurs when secretion is inhibited (Meschi et al., 2019; Agbu et al., 2020). We conclude that the knockdown of EGFR activity at the semi-permissive temperature of 26.5°C has no significant effect on dILP2 secretion and therefore EGFR is not indirectly acting on Yan through this process.

The extinction dynamics of Yan, which the model predicts to be similar under reduced metabolism conditions (Fig. 3D), appear to be slower under EGFR mutant conditions (Fig. 3E,F). The shaded regions shown for the experimental data correspond to 95% confidence intervals for the moving average Yan-YFP level as a function of developmental time. These confidence intervals were estimated by re-sampling both independent eye disks and the cells within them, then computing the moving average at each time point for each sample. Thus, the shaded region is a measurement statistic that reflects the variance of the estimate for the average trajectory. In contrast, the variance among independent simulation trajectories is a population statistic that reflects the expected spread among the entire population of potential trajectories. Because the two types of intervals measure different things, it is not expected that the trends qualitatively match between model and experiment.

**Fig. 4.**
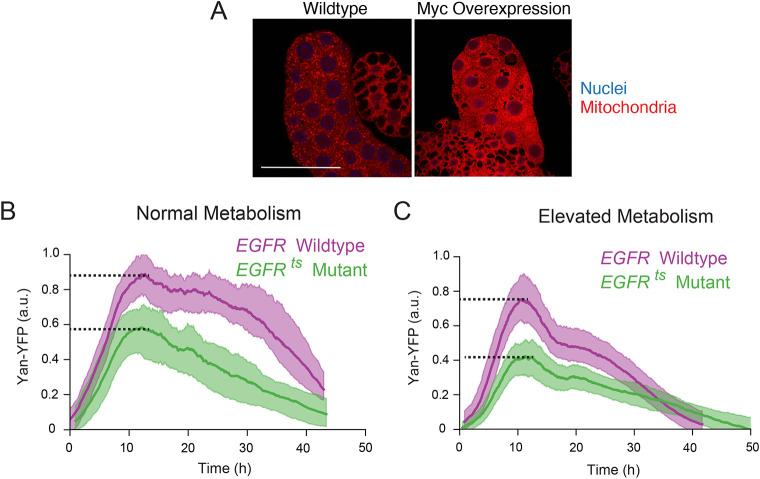
**Yan expression is more dependent on EGFR when metabolism is elevated.** (A) Larval salivary gland cells from *ptc-Gal4/+* (left) and *ptc-Gal4/UAS-Myc* (right) animals. Tissue was stained for active mitochondria (red) and nuclei (blue). Note the higher density of mitochondria in cells overexpressing Myc. Scale bar: 25 μm. (B,C) Yan-YFP expression dynamics in eye disk progenitor cells that are wild-type *EGFR* (*EGFR^tsla^/EGFR^+^*) or mutant *EGFR* (*EGFR^tsla^/EGFR^f24^*) raised at the semi-permissive temperature. Time 0 marks the time at which Yan expression begins. Purple and green solid lines are moving averages of wild-type and mutant cells, respectively. Shaded regions denote 95% confidence intervals. Each line average is calculated from a composite of measurements of between 4448 and 8653 cells. (B) Yan-YFP dynamics under normal (*GMR-Gal4/+*) metabolic conditions. (C) Yan-YFP dynamics in *GMR-Gal4/UAS-Myc* eye disks. Although it appears that overall Yan-YFP output is not increased with Myc overexpression, it is important to keep in mind that Yan repressors are also likely more active, contributing to a more normalized expression output. Dotted lines indicate peak Yan-YFP levels a.u., arbitrary units.

Our model also predicted that elevating metabolism to above-normal levels would enhance the dependence of gene expression on activators (Figs 1G and 2H). To test this prediction, we overexpressed the transcription factor Myc in eye cells. This induces elevated cellular growth and reconfigures cellular metabolism so that oxidative phosphorylation is displaced by aerobic glycolysis (Dang, 1999; Johnston et al., 1999; Secombe et al., 2007; de la Cova et al., 2014). There is an apparent increase in metabolic rate because glucose consumption is increased in wing disk cells that overexpress Myc (de la Cova et al., 2014). Myc overexpression also increases mitochondria density in cells (de la Cova et al., 2014), which we observed when overexpressing Myc in *Drosophila* cells (Fig. 4A).

We specifically overexpressed Myc in eye epithelial cells (Fig. 4B,C). A pulse of Yan expression occurred although there appeared to be a sharper decline in Yan abundance over time. This might be due to enhanced protein and mRNA turnover in the hyper-growing cells. When we compared the effect of the *EGFR* mutant in Myc-expressing cells, we observed a greater impact of EGFR on Yan output. Peak expression was reduced by 34% in a wild-type Myc background, (Fig. 4B), and by 45% in the Myc overexpression background (Fig. 4C). Thus, modulating metabolic activity to be lower or higher than normal demonstrates how activators have variable effects on target gene expression depending on metabolic conditions.

### Developmental outcomes are dependent on metabolism–gene activator interactions

**Fig. 5.**
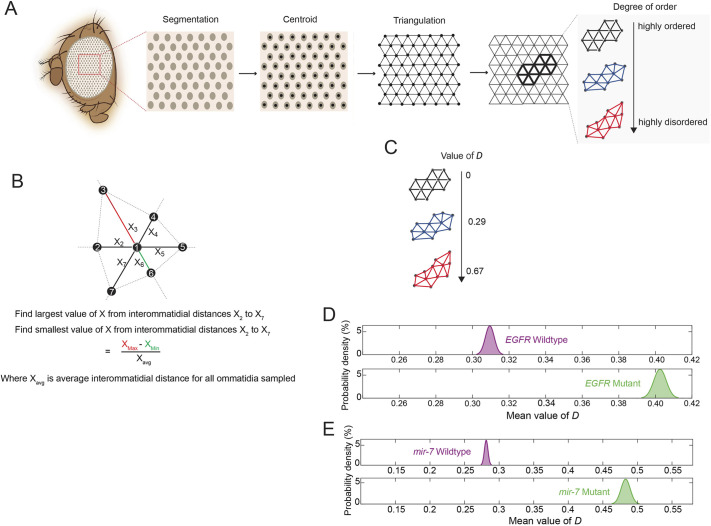
**Quantification of lattice disorder in mutant compound eyes.** (A) Pipeline of analysis involving segmentation of ommatidia, triangulation of their centroids to recreate the overall lattice, and local lattice analysis of every ommatidium and its nearest neighbors. (B) Local lattice disorder is estimated as the difference between the longest distance from one ommatidium to a nearest neighbor and the shortest distance from that ommatidium to a nearest neighbor. This normalized difference *D* is calculated for every ommatidium in the region of interest. (C) Schematic of ommatidia with varying values of *D* and therefore varying levels of disorder. (D) Density distributions of the mean *D* estimated for ommatidia from wild-type (*EGFR^tsla^/EGFR^+^*) (purple) and *EGFR ts* mutant (*EGFR^tsla^/EGFR^f24^*) (green) eyes. Animals were raised at 18°C except for an 18-h interval as late L3 larvae when they were incubated at 26.5°C. The numbers of ommatidia analyzed for each dataset were 1321 and 1438, respectively, and these were each imaged from ten animals. (E) Density distributions of the mean *D* estimated for ommatidia from wild-type (purple) and *mir-7* mutant (green) eyes. The numbers of ommatidia analyzed for each dataset were 1333 and 1130, respectively, and these were each imaged from ten animals.

We had previously found that ablation of IPCs suppressed developmental phenotypes caused by mutations in gene repressors (Cassidy et al., 2019). Therefore, we wondered whether IPC ablation also suppressed phenotypes caused by loss of activators. Yan is important for the proper specification of photoreceptors in the eye (Voas and Rebay, 2004), and transient loss of EGFR activity results in mispatterned adult compound eyes in which the highly regular hexagonal lattice of unit eyes (ommatidia) becomes disordered (Peláez et al., 2015; Kumar et al., 1998). To measure precisely the degree of disorder in such eyes, we developed a new image-based analysis pipeline. Brightfield microscopy of adult compound eyes captured the reflection points of individual ommatidium lenses (Fig. S5A-E). After computational segmentation of reflection points, triangulation of their centroids allowed us to measure the distance between each ommatidium and all its immediately adjacent neighbors (Fig. 5A, Fig. S5F-I). For each ommatidium, we calculated the difference between its distance to its closest neighbor and its distance to its farthest neighbor. This value, *D*, normalized by the average interommatidial distance, was calculated for all ommatidia in all eye samples (Fig. 5B). The *D* metric is a measure of lattice disorder. Errorless measurements on a perfectly regular lattice yield an average *D* of zero; measurement errors on the order of 10% of the average distance yield a *D*≈0.15. Disordered lattices will yield even higher values for *D* (Fig. 5C). We applied the method to measure disorder in the compound eyes of wild-type and mutant flies. EGFR activates and the miRNA miR-7 represses expression of Yan in the eye (Peláez et al., 2015; Li and Carthew, 2005). Visual inspection of *EGFR* and *mir-7* mutant eyes showed a qualitatively greater disorder compared with wild type (Fig. S6). Quantitatively, both the *EGFR* and *mir-7* mutant adults exhibited greater eye lattice disorder as their mean values for the *D* metric were significantly higher than wild-type controls (Fig. 5D,E).

**Fig. 6.**
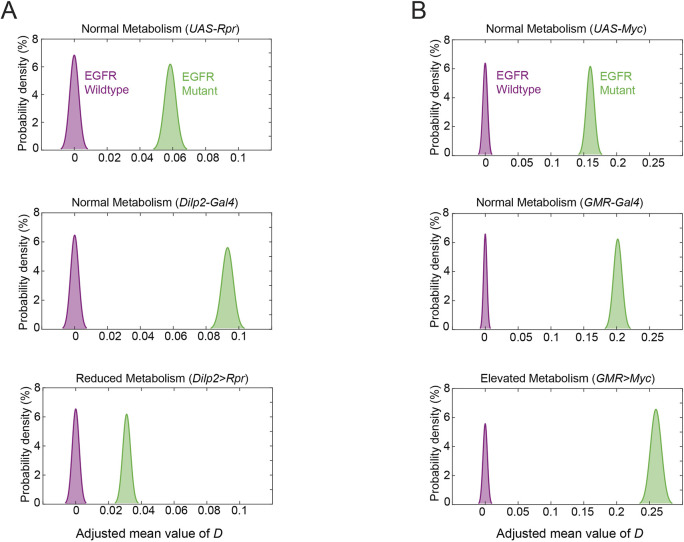
**EGFR loss affects disorder of the eye lattice dependent on energy metabolism.** (A,B) To compare *EGFR* genotypes, the density distribution of the mean *D* estimated for ommatidia from wild-type *EGFR* eyes (purple) was adjusted to center around zero, and the *EGFR* mutant distribution (green) was normalized accordingly. (A) Disorder of *EGFR* mutants when metabolism is normal or is reduced by IPC ablation. (B) Disorder of *EGFR* mutants when metabolism is normal or is elevated by Myc overexpression.

We then measured eye lattice disorder in *EGFR* mutants in which IPCs had been ablated (Fig. 6A, Fig. S7). The mean *D* metric was significantly lower in *EGFR* mutants with IPC ablation than in *EGFR* mutants with normal metabolism. Hence, lowering energy metabolism suppresses the developmental phenotype of the *EGFR* mutant. We also measured disorder in *EGFR* mutants that overexpressed Myc in eye cells (Fig. 6B, Fig. S7). There was a significant increase in the mean *D* metric relative to controls with normal metabolism. Thus, raising metabolism enhances the *EGFR* developmental phenotype. Overall, these results are consistent with the results of Yan expression experiments, implicating energy metabolism as a modulator of gene regulation through activation.

## DISCUSSION

Development and growth are fueled by energy metabolism, suggesting that the tempo of development depends on metabolic rate. Thus, the dynamics of developmental GRNs must faithfully adjust to a variable time scale. Previously, we found that auxiliary repressors of gene expression become non-essential when metabolism is decreased (Cassidy et al., 2019). However, it was unclear whether auxiliary activators are also non-essential when metabolism is decreased. Moreover, what are the effects of gene regulators when metabolism is increased above the normal level? In this study, we have shown that auxiliary gene activators have differential effects on target gene expression when metabolism is set to above- or below-normal conditions. Activation by EGFR is required for proper Yan expression and developmental outcome when energy metabolism is normal, and EGFR becomes functionally redundant when metabolism is lowered. EGFR becomes more necessary for proper developmental outcome when metabolism is increased above normal. A stylized mathematical model predicts that this relationship between gene activators and below-normal and above-normal metabolism is not limited to Yan expression but exists for many genes and their activation factors. In this way, auxiliary gene activation allows for reliable development across a much broader range of metabolic conditions than would otherwise be tolerated.

The intuition for understanding this relationship resides in the fact that limiting ATP turnover reduces the rates of reactions, and the resulting slower dynamics of synthesis and degradation allow for weaker activation to nevertheless achieve peak output. A different interpretation of our findings has to do with the fact that gene expression is inherently stochastic, a feature that our model also incorporates. In our modeling, the relative error in gene expression (signal-to-noise ratio) scales with peak expression levels such that higher peak expression is related to lower relative error. Thus, when metabolism is reduced and peak expression is consequently lower, the relative error is increased. This results in more overlap between the distribution of gene expression with full activation and the distribution with partial activation. The enhanced overlap can be viewed as greater similarity between full and partial activation when metabolism is reduced. In contrast, enhancing metabolism decreases relative error, resulting in less overlap in the two distributions, and, therefore, less similarity between full and partial activation.

Developmental tempo and metabolic rates vary with temperature, making it possible that temperature-dependent gene expression dynamics are coupled to a metabolism-dependent time scale. We have explored this notion using our modeling framework, and the results are inconclusive (S.B., N.B. and L.A.N.A., unpublished results).

Our model is not limited to developmental genes but could be relevant for any genes that are expressed with pulsatile dynamics. Many genes in both unicellular and multicellular organisms have these features (Levine et al., 2013; Dalal et al., 2014). Examples include stress response genes, and genes involved in signal transduction (Hansen and O'Shea, 2016; Hafner et al., 2017). It will be interesting to discover whether our model predictions of metabolism and gene control extend to such situations.

The insect eye is an example of a system with remarkable spatial order that is driven by a demand for optimal visual acuity and sensitivity (Nilsson, 1989; Land, 1997). We have developed an imaging-based analysis tool that precisely measures spatial order in the *Drosophila* compound eye. It is sufficiently sensitive to detect differences in spatial order due to genetic background. It is amenable to population averaging and statistics. The method is readily adaptable for general use and is potentially applicable for insect species other than *Drosophila melanogaster*. Here, we apply it to measure disorder in genetic mutants, but it can be used to study the effects of other perturbations, and its sensitivity will potentially be useful for even the weakest of perturbations.

## MATERIALS AND METHODS

### *Drosophila* growth and genetics

For all experiments, *Drosophila melanogaster* was raised using standard lab conditions and food. All experiments used female animals unless stated otherwise. Stocks were either obtained from the Bloomington *Drosophila* Stock Center, from listed labs, or were derived in the laboratory of R.W.C. Experiments with *EGFR* were performed using trans-heterozygous mutants in order to minimize phenotypes induced by secondary mutations on relevant chromosomes. Trans-heterozygous allele combinations used were *Egfr^tsla^*/*Egfr^f24^* (Peláez et al., 2015). Genetic mosaic animals bearing *mir-7*^△*1*^ homozygous mutant eyes were generated using the ey-FLP/FRT system as described (Cassidy et al., 2019).

The BAC genomic transgene *Yan-YFP* was inserted on chromosome 3 at the attP2 site. The *Yan-YFP* chromosome was homozygosed so that animals had two copies of the transgene, and placed in a *yan^443^/yan^884^* mutant background so that the endogenous *yan* gene did not make any protein.

To genetically ablate the IPCs of the brain, *yw* animals were constructed bearing a *dILP2-Gal4* transgene on chromosome 3 and a *UAS-Reaper* (*rpr*) transgene on chromosome 1. *rpr* is a pro-apoptotic gene that is sufficient to kill cells in which it is expressed (Lohmann et al., 2002). *dILP2-Gal4* fuses the *Insulin-like peptide 2* gene promoter to Gal4, and specifically drives its expression in brain IPCs (Rulifson et al., 2002). Examination of *dILP2-Gal4; UAS-Rpr* larval brains showed that they almost completely lacked IPCs (Cassidy et al., 2019). Previous studies found that IPC-deficient adults are normally proportioned but of smaller size. It takes 70% longer to complete juvenile development, and juveniles have a 40% elevation in blood glucose, consistent with dILPs being essential regulators of glucose metabolism in *Drosophila* (Cassidy et al., 2019; Rulifson et al., 2002; Ikeya et al., 2002; Broughton et al., 2005). Moreover, animals generate 30% less heat output as measured by whole-body calorimetry (Zhang et al., 2009), and ATP synthase abundance is reduced in cells from IPC-ablated larvae (Cassidy et al., 2019). For all wild-type controls, we tested animals bearing either the *dILP2-Gal4* or *UAS-Rpr* gene in their genomes.

To overexpress the *Drosophila* transcription factor Myc in cells, animals were constructed with a *UAS-Myc.Z* transgene located on chromosome 2 or 3. This transgene was activated using either a *GMR-Gal4* transgene or *ptc-Gal4* transgene located on chromosome 3 or 2. *GMR-Gal4* drives UAS gene expression in all larval eye cells posterior to the morphogenetic furrow (Freeman, 1996), whereas *ptc-Gal4* drives UAS gene expression in larval salivary gland cells (Aza-Blanc et al., 1997). Ptc>Myc causes salivary gland cells to grow faster owing to enhanced translation capacity (Grewal et al., 2005). GMR>Myc causes eye cells to grow 33% bigger (Secombe et al., 2007). Myc overexpression reconfigures cellular metabolism in imaginal disks so that oxidative phosphorylation is displaced as the predominant source of energy production by increased glycolysis, resembling the Warburg effect (de la Cova et al., 2014). Expression of Myc increased the mitochondrial network, consistent with Myc's regulation of mitochondrial biogenesis (de la Cova et al., 2014).

EGFR activity was conditionally reduced by placing *Egfr^f24^* (Clifford and Schüpbach, 1989) *in trans* to the ts mutant allele *Egfr^tsla^* (Kumar et al., 1998). Flies were raised at the permissive temperature (18°C) and shifted to a semi-restrictive temperature (26.5°C) as third instar larvae for 18 h. Genetic wild-type controls were heterozygotes of either *Egfr^f24^* or *Egfr^tsla^* over a wild-type chromosome. These controls were also shifted to 26.5°C as third instar larvae for 18 h. At 26.5°C, developing eye cells had compromised EGFR activity in *Egfr^tsla^*/*Egfr^f24^* animals as when transferred back to the permissive temperature and allowed to eclose, they had rough eyes (Peláez et al., 2015). In mutant eye disks, there were signs of some cells undergoing apoptosis: a significant reduction of nuclear diameter, a strong Yan-YFP brightness, and anomalous nuclear position along the apical-basal axis. Apoptosis was more prevalent in disks from mutant animals treated at temperatures greater than 26.5°C (Peláez et al., 2015). Therefore, we chose this temperature for EGFR activity reduction so as to minimize apoptosis but still achieve effects on Yan expression. We only included in our analysis cells corresponding to classical anatomical positions and apical basal migration patterns.

### Quantification of Yan-YFP expression dynamics in the eye

Eye disks from white-prepupae of the correct genotype (*yan^443^/yan^884^; Yan-YFP/Yan-YFP*) were dissected, fixed and imaged by confocal microscopy, as previously described (Peláez et al., 2015; Bernasek et al., 2023). These prepupae also had the appropriate combinations of *Egfr* alleles, *Gal4* driver genes, and *UAS* transgenes for controlled manipulation of EGFR activity and cell metabolism. Eye disks were fixed for 45 min at room temperature in 4% (w/v) paraformaldehyde in PBS. Disks were washed in PBS and then incubated in 1:1 (v/v) PBS:VECTASHIELD with DAPI (Vector Laboratories) for 45 min, followed by a 45 min incubation in 100% VECTASHIELD with DAPI. Samples were then mounted with VECTASHIELD with DAPI and imaged using a Leica TCS SP8 confocal microscope equipped with a 40× oil objective (NA=1.3) with a digital zoom of 1.2-1.4. Yan-YFP and DAPI were separately detected by HyD detectors (GaAsP). During imaging, disks were oriented with the morphogenetic furrow parallel to the *y*-axis of the image. Optical slices were captured as 1024×1024 8-bit images, in which at least six rows of ommatidia on either side of the dorsal-ventral equator were recorded. Optical slices were set at 0.7 μm thickness, and 45-60 optical slices were captured in a *z*-stack to completely image eye disks from basal to apical surfaces. All disks for a given condition were fixed, mounted and imaged in parallel to reduce measurement error. Sample preparation, imaging and analysis were not performed by operators who were aware of the sample groupings. See Fig. S3 for examples of typical imaging data.

Image data were processed for automatic segmentation and quantification of DAPI and YFP nuclear fluorescence as described (Peláez et al., 2015; Bernasek et al., 2023). Briefly, cell segmentation was performed using the DAPI signal as a reference channel for identification of the boundaries of cell nuclei. Each layer of the reference channel was segmented independently. A single contour containing each unique cell was manually selected and assigned a cell type using a custom graphic user interface called Silhouette. For each annotated cell contour, expression measurements were obtained by normalizing the mean pixel fluorescence of the YFP channel to the mean fluorescence of the DAPI channel. This normalization serves to mitigate variability due to potentially uneven sample illumination, segment area, and differences in protein expression capacity between cells. We assigned cell-type identities to segmented nuclei by using nuclear position and morphology, two key features that enable one to identify eye cell types unambiguously without the need for cell-specific markers (Wolff and Ready, 1993). This task was accomplished using Silhouette, an open-source package for macOS that integrates our image segmentation algorithm with a graphical user interface (GUI) for cell-type annotation. Subsequent analysis and visualization procedures were implemented in Python using the FlyEye package, open source software developed by our group.

Using FlyEye, cell positions along the anterior-posterior axis were mapped to developmental time as described previously (Peláez et al., 2015; Bernasek et al., 2023). This is predicated on two assumptions: (1) the furrow proceeds at a constant rate of one column of R8 neurons per 2 h; and (2) minimal cell migration occurs. For each disk, Delaunay triangulations were used to estimate the median distance between adjacent columns of R8 neurons (Fortune, 1992). We used the median rather than the mean distance because it minimized the influence of non-adjacent R8s that were falsely identified by the triangulation (Peláez et al., 2015). Dividing the furrow velocity of 2 h per column by this median distance yields a single conversion factor from position along the anterior-posterior axis to developmental time. This factor was applied to all cell measurements within the corresponding disk, yielding expression time series. Notably, these are not single cell dynamics, but rather aggregate dynamics across the development time course of a spatially organized cell population.

Moving averages were computed by first-order Savitzky–Golay filtration (Savitzky and Golay, 1964). This method augments the simple windowing approach used in by Peláez et al. (2015) by enabling visualization of expression trends at early time points that are otherwise obscured by large window sizes. A secondary first-order filtration with one-fifth the original window size was applied to smooth lines for visualization purposes. None of our conclusions was sensitive to our choice of filtration or smoothing method. A primary window size of 250 cells was used for reporting the expression of cells, unless noted otherwise. Confidence intervals for the moving average were inferred from the 2.5th and 97.5th percentile of 1000-point estimates of the mean within each window. Point estimates were generated by bootstrap resampling with replacement of the expression levels within each window.

To align multiple eye disk samples using FlyEye, cells of each sample were aligned with a reference population by shifting them in time as described by Bernasek et al. (2023). The magnitude of this shift was determined by maximizing the cross-correlation of progenitor Yan-YFP expression *Y(t)* with the corresponding reference time series *X(t)*. Rather than raw measurements, moving averages within a window of ten cells were used to improve robustness against noise. This operation amounts to:
(1)

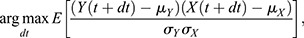
where, *μ* and *σ* are the mean and standard deviation of each time series, and *dt* is the time shift by which the population should be shifted.

For each experimental treatment, a disk was randomly chosen and shifted in time such that time ‘zero’ corresponds to the first annotated R8 neuron. This disk then served as the reference population for the alignment of all subsequent biological replicates within the treatment. Similarly, different experimental treatments (e.g. control and perturbation) were aligned by first aligning the disks within each treatment, then aggregating all cells within each treatment and repeating the procedure with the first treatment serving as the reference. To plot the moving line averages of each aggregate dataset, we adjusted time on the *x*-axis such that −10 h became the new 0 h and all other time intervals were adjusted accordingly. Yan-YFP was first detectable in cells no earlier than the −10 h time point.

We analyzed 6-12 replicate eye discs for each treatment in two separate experiments. In total, we measured 5406 and 4870 cells under normal metabolism for *EGFR* wild-type and *EGFR* mutant samples, respectively. We measured 4448 and 5186 cells from *Dilp2>Rpr* animals for *EGFR* wild-type and *EGFR* mutant samples, respectively. We measured 4668 and 8853 cells from *GMR>Myc* animals for *EGFR* wild-type and *EGFR* mutant samples, respectively.

### Immunohistochemistry of dILP2 in IPCs

Larvae of the genotypes *Egfr^tsla^/Egfr^f24^* or *Egfr^tsla^/Egfr^+^* were raised at 18°C and then shifted to 26.5°C for 16 h before harvesting. Larval brains were fixed in 4% (w/v) paraformaldehyde for 30 min at room temperature (RT), washed with PBS containing 0.1% Triton X-100 (PBST) three times for 15 min each, and blocked at RT using PBST+5% (v/v) bovine serum albumin (BSA) for 2 h. Samples were incubated in primary antibody (1:400; rat anti-dILP2, gift of P. Leopold) (Meschi et al., 2019) diluted in PBST+5% BSA at 4°C overnight. Brains were washed in PBST three times for 5 min each then incubated in secondary antibody (1:500; Alexa Fluor 647 goat anti-rat, Thermo Fisher Scientific, A21247) diluted in PBST+5% BSA for 2 h at RT. After washing three times for 5 min each with PBST, brains were mounted in VECTASHIELD. Samples were imaged using a 40× oil immersion objective on a Leica SP8 laser-scanning confocal microscope. dILP2 fluorescence signal was excited with a 2% powered 638 nm laser and captured by using a HyD detector with 100% gain, with each *z*-step size being 0.5 μm/slice in a 512×512 µm *xy* field of view. Microscopy parameters were kept constant for both the heterozygous controls and mutants, and all imaging was performed in one session. Image analysis was performed using Fiji. Average fluorescence intensity was measured for multiple IPC termini, where dILP2 is stored prior to secretion. These measurements were corrected by subtracting the background fluorescent intensity in the images.

### Imaging of adult compound eyes

For imaging compound eyes, 2- to 3-day-old adults of different genotypes were collected and stored in 100% ethanol. Before imaging, samples were progressively rehydrated by successive 24-h incubations in 75% ethanol, 50% ethanol, 25% ethanol, and water. Blu Tack (Bostik Smart Adhesives) was cut into a 1.5 cm piece and pressed using a thumb onto a microscope slide. The rehydrated flies were transferred onto a Kimwipe to briefly dry, and then were placed laterally onto the Blu Tack with their left eyes facing up and oriented horizontally (Fig. S5A). Mounted animals were imaged with a Leica DM6B bright-field microscope with a 10× objective (NA=0.40) and DFC7000T camera. To illuminate the samples, gooseneck fiber-optic lights (Schott, KL 1500 LCD) were positioned above the stage on opposing sides of the specimen. The two lights were positioned facing one another, and the angle of the fiber-optic cables was parallel to the stage (Fig. S5B). Because image quality is affected by positioning of the light source, a criterion for a good quality image is determined by avoiding (1) uneven distribution of the light, (2) double reflective images from each ommatidium, and (3) reflection from non-ommatidia regions (Fig. S5D,E). All objectives except the one in use must be removed from the microscope to prevent these lighting/imaging aberrations. In addition, the gooseneck lights must be subtly adjusted for each sample to avoid lighting aberrations. Exposure time was adjusted according to the eye color and eye size of each sample to ensure a uniform field of reflective points. An example of a high-quality image is shown in Fig. S5C. Optical slices were captured at 10-μm intervals along the *z*-plane using the Leica Application Suite X. The stack of raw image files for each sample was imported into Zerene Stacker (Zerene Systems), from which DMAP images were generated as described (Iyer et al., 2016). Zerene Stacker projects the entire stack into a DMAP image. The DMAP files from Zerene Stacker were then used for further analysis.

### Pipeline for quantification of eye disorder

To computationally segment ommatidia, the raw DMAP images were pixel-classified into two classes of pixels: (1) pixels representing light reflected from ommatidia (identified by the reflection from each ommatidia lens), and (2) pixels representing all other features of the images. This pixel classification step was achieved using the ‘Pixel Classification’ workflow in Ilastik, which is open-source software that provides machine-learning image analysis (Berg et al., 2019). Briefly, Ilastik was trained on 12 DMAP images from six different genotypes (two images were chosen from each genotype). This was to ensure good representation of the variability contained within the complete dataset. For the 12 images, pixels were manually annotated as belonging to the light reflected from ommatidia or not. This process was repeated until the model learned to classify pixels satisfactorily, determined using the live prediction feature of Ilastik. Once a satisfactory model was trained, the remaining 48 images in each dataset were pixel-classified using the trained Ilastik model.

Custom MATLAB scripts were developed that (1) import the pixel classification maps generated by the model trained in Ilastik, (2) threshold the pixel classification maps to obtain binary maps where 1 represents an ommatidia light reflection, and 0 represents all other pixels, (3) detect all isolated binary objects (contiguous pixels of value 1 that correspond to each ommatidium), and (4) compute the centroid of each binary object, which then becomes the defined center of each ommatidium. Because each binary object was typically 10-20 pixels in size, it necessitated calculation of the object's centroid to have a single pixel that defines the location of each ommatidium.

Because ommatidia were detected via light reflections, the automated workflow led to some misclassification of other reflections as originating from ommatidia. These reflections often occurred at ommatidial boundaries (particularly for rough eye phenotypes) and outside the eye field on the cuticle of the head. Therefore, a custom GUI was developed in MATLAB that allowed for manual correction of the data derived from the above-described workflow. Briefly, this GUI displayed the DMAP images overlaid with all identified centroids, allowing the user to add new centroids for ommatidia that were not classified as ommatidia. It also allows the user to delete centroids that were improperly classified as ommatidia. This GUI was used to thoroughly correct the classification of ommatidia from the data.

For each analyzed image, the ommatidial lattice was defined using Delaunay triangulation of the centroids, implemented using a built-in MATLAB function. Because a triangular lattice is dual to a hexagonal lattice, Delaunay triangulation allows for analysis of the component triangles of each hexagonal unit of the compound eye, i.e. for wild-type eyes, each ommatidial unit is composed of six triangles that define the space between one ommatidia and its six closest neighbors; together, these six triangles create one hexagonal unit.

Interommatidial distances were calculated for all ommatidia except for those along the boundary of the region of analysis. This is because ommatidia along the boundary had neighbors that were not identified, preventing proper calculation of their local disorder. Boundary ommatidia did, however, contribute to the calculation of local disorder for ommatidia to which they were neighbors. Boundary ommatidia were identified by finding the convex hull of segmented ommatidia using the built-in MATLAB function.

Because we had generated a 2D image of a 3D curved structure, there were inherent distortions of the derived lattice that contributed to our estimation of lattice disorder. To minimize this curvature-based distortion of interommatidial distances, we only analyzed ommatidia within a fixed distance from the center of each eye, where there was minimal curvature. The distance chosen was 200 pixels and the center of each eye was estimated as the center of mass of segmented ommatidia. By limiting the analysis to a small region of the eye, effects of curvature were minimized.

Definition of the ommatidial lattice by Delaunay triangulation creates several easy-to-measure features of lattice order. These features include (1) the length of each triangle edge, (2) the area of each triangle, and (3) the angles formed at the vertices of each triangle. In a perfectly regular lattice, each of these three features would be uniform, i.e. all sides of every triangle would be the exact same length. We initially calculated lattice regularity by analyzing the distributions of the aforementioned lattice features. Variation was estimated as either the coefficient of variation or the Fano factor. Although these estimates are descriptive of lattice order/disorder, they are not sensitive to infrequent or mild lattice defects.

Therefore, we devised a more sensitive measurement of lattice order. Local regularity of the lattice was defined as the variability in the distances that connect one ommatidium to its nearest neighbors. By finding the largest (*X_max_*) and smallest (*X_min_*) distance connecting one ommatidium to its nearest neighbors, the difference in those two distances can be calculated as a measure of local order. For example, if *X_max_*−*X_min_* is zero, then the local lattice has perfect order. The larger the value for this difference, the greater the local disorder. We then normalized the difference to estimate:
(2)

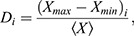
where *D_i_* is the degree of disorder for ommatidium *i*, and <*X*> is the mean distance between every ommatidium and its nearest neighbors. This *D* metric was calculated for every ommatidium analyzed in all eye samples from a given condition.

Another benefit of analyzing the regularity of the lattice on such a local scale is that it controls for distortion from eye curvature of the eye. When we calculated lattice order using the aforementioned three lattice features described above, a large contributor to the variability came from curvature-based distortion. As *D_i_* is a local measurement, the scale of curvature is much greater than the scale of the measurement. Therefore, curvature contributes much less to the variability of *D_i_* measurements.

For statistical analysis, we performed bootstrapping on the thousands of measurements of *D* for each genetic condition (ranging from 993 to 1473). Bootstrapping was performed in MATLAB 10,000 times, and the mean value of *D* was calculated per bootstrap sample. The distribution of means was plotted as a histogram, and shown in the figures are the smoothed fits to each of the histograms.

The complete computational pipeline for segmentation, correction, and analysis that is described above is freely available as a MATLAB software package called roughEye.

### Mitochondria staining

Second instar larvae (either *ptc-Gal4* genotype or *ptc-Gal4/UAS-Myc* genotype) were dissected, and salivary glands were incubated in PBS supplemented with 500 nM MitoTracker Red CMXRos (Thermo Fisher Scientific) for 30 min at room temperature. This reagent localizes to mitochondia, and its fluorescence depends upon the membrane potential found in active mitochondria. Glands were then washed several times with Schneiders medium for 10 min at room temperature. Glands were fixed in 4% (w/v) paraformaldehyde in PBS for 20 min. After washing in PBS, glands were mounted in VECTASHIELD (Vector Laboratories) with DAPI (to visualize nuclei). The samples were imaged with a Leica SP5 confocal microscopy system.

### Mathematical modeling

Our modeling framework is based on the one we developed previously (Cassidy et al., 2019). It directly describes the emergent expression dynamics of a single gene within a cascade of developmental gene expression. It leverages two key concepts from control theory. The first is the notion of Lyapunov stability; that is, systems tend to remain near stable equilibria. The second is the Hartman–Grobman theorem, which posits that systems deviate approximately linearly about these fixed points (Arrowsmith and Place, 1992). We therefore developed a model that describes the time evolution of linear deviations about the basal protein level that exists before gene expression is induced and after it subsides.

Specifically, a linear time-invariant system describes the time evolution of deviations (Δ) in activated DNA (Δ*D*), mRNA (Δ*R*) and protein (Δ*P*) state variables in response to a change in stimulus (Δ*I*) that induces gene activation. These discrete state variables depict the extent to which gene expression has deviated from its basal level at any point in time. Transitions between each of the variables' states are governed by the stochastic processes listed given in Table 1.

**Table 1.**
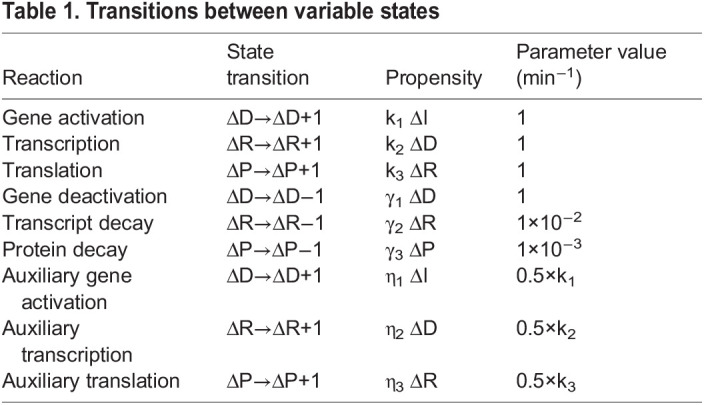
Transitions between variable states

In the continuum limit, this model yields a deterministic system of differential equations:
(3)

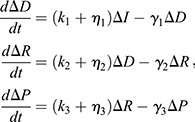
where *k_i_* are activation, transcription or translation rate constants, *η_i_* are their auxiliary counterparts, and *γ_i_* are degradation constants. In control parlance, three sequential first-order transfer functions relate input disturbances to deviations in output protein level.

The key distinction between this model and the model used in our previous study is that we have replaced the auxiliary repression terms with auxiliary activation terms (*η_i_*). The models are otherwise the same, and still include the linear degradation kinetics (*γ_i_*) required to drive the system back to basal levels once the transient input subsides. We do not expect that including auxiliary repression terms equivalent to those in our previous study would yield any qualitative changes in the results or conclusions presented here.

### Dependence of model parameters on metabolic conditions

IPC ablation reduces cellular glucose consumption. Presumably, this would affect the production and consumption of ATP. Given that ATP concentration remains fairly constant when respiration is limited (Brown, 1992), ATP flux (and ATP synthesis) is assumed to decrease. Because transcription, translation and protein degradation all require ATP turnover, we halved their rate parameters under conditions of reduced glucose consumption. All auxiliary activator strengths were reduced in an equivalent manner to their primary counterparts. That is, rate constants for auxiliary activators driving either transcription or translation were each halved under conditions of reduced glucose consumption. These assumptions are incorporated as changes to the model's rate parameters as listed in Table 2. Conversely, to model the effects of increased ATP consumption we instead increased each of the relevant rate parameters by 50%.

**Table 2.**
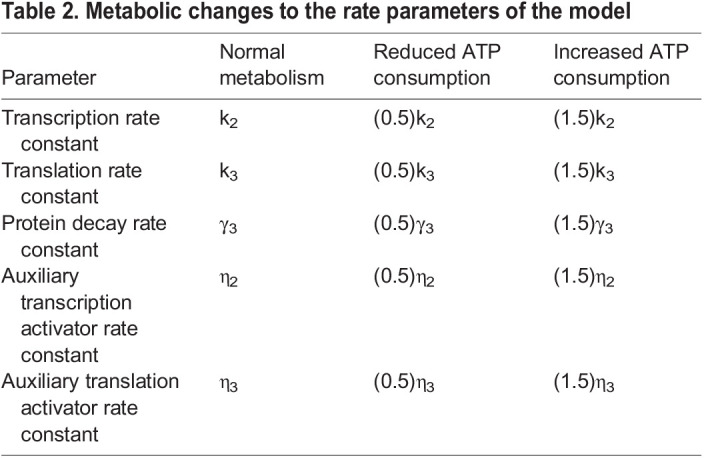
Metabolic changes to the rate parameters of the model

### Model simulations

Default parameter values were based on approximate transcript and protein synthesis and turnover rates for animal cells reported in the literature (Milo and Phillips, 2015), whereas gene activation and decay rates were arbitrarily set to a significantly faster time scale. Default strengths for auxiliary activators acting at the gene, transcript or protein levels were chosen such that ∼60% of simulations failed to reach the threshold protein level under normal conditions when the auxiliary activator was lost. Population-wide expression dynamics were estimated by simulating 5000 output trajectories in response to a 3-hour transient step input to the gene activation rate. Simulations were performed using a custom implementation of the stochastic simulation algorithm (Gillespie, 1977). The algorithm constrains solutions to the set of discrete positive values, consistent with linearization about a basal level of zero gene activity. This simplifying assumption is based on the near-zero basal activities expected in the experimental systems, but is not required to support the conclusions of the model (Figs S2C and S4C).

### Evaluation of error frequencies and changes in expression dynamics

Gene expression trajectories were simulated both with (full activation) and without (partial activation) auxiliary activators. Protein expression dynamics were compared by evaluating the fraction of partial-activation simulation trajectories that fell below the top 99% of full-activation trajectories at each point in time, *t*. We refer to this under-expression value as *E(t)*. The time point at which the full-activation simulations mean level reached 30% of its maximum value was taken to be the commitment time. Under-expression was averaged across the time course, beginning with the reception of the input and ending at the commitment time, *τ*:
(4)

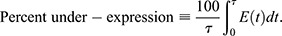
Percent under-expression reflects the net extent to which the expression dynamics differ between the two sets of simulated trajectories.

To estimate the error frequency due to loss of auxiliary activators, the instantaneous error rate was computed by evaluating *E(t)* at the time at which the full-activation simulations mean level reached its maximum amplitude. Because the threshold was set at the bottom 1% of full-activation protein levels, the minimum possible error frequency is 1%. For simplicity, we subtracted this percentage point from all reported error frequencies.

This definition of error frequency differs from that used in our prior study in that it quantifies the frequency of failure to reach a minimum required level for successful development, rather than the frequency of failure to attenuate expression below a maximum allowable level. This change was made in order to define a framework for evaluating the influence of activators of gene expression, which are generally responsible for increasing target protein levels.

### Parameter variation and sensitivity to model assumptions

We conducted parameter sweeps to confirm the robustness of each computational result. In each sweep, all model parameters were varied across a tenfold range (± ∼threefold). We quasi-randomly generated 1000 such parameter sets, then independently ran four sets of 5000 simulations for each: (1) full activation with normal metabolism, (2) partial activation with normal metabolism, (3) full activation with reduced metabolism, and (4) partial activation with reduced metabolism. Partial-activation systems were assigned a single primary activator for each stage of synthesis. In addition to these primary activators, each full-activation system was assigned an additional set of auxiliary activators the strengths of which relative to the primary activators were specified by a free parameter we refer to as ‘severity’. Error frequencies were evaluated as described above.

Each sweep sampled a seven-dimensional space. Projecting the results of all simulations onto each of the 21 orthogonal 2D planes revealed that error frequency is greater than 1% for almost all combinations of parameter values (Fig. S1A). Although it helps illustrate our parameter sweep methodology, the 2D visualization does not offer sufficient insight into the global trend to justify its complexity. We instead opted for a condensed 1D projection (Fig. S1B), which clearly indicated that loss of auxiliary activators induces an increase in error frequency across a broad parameter range. Auxiliary activator loss also decreased protein levels throughout the time course for the vast majority of parameter sets (Fig. S3A).

The difference in error frequency between simulations with normal metabolism and reduced metabolism are shown in Figs S1C and S2 for all parameter sets. There was a general trend of decreased error frequency with partial activation under reduced energy metabolism conditions. The difference in protein under-expression between simulations with normal versus reduced metabolism are shown for all parameter sets in Figs S3B and S4. Most parameter sets showed less under-expression in the absence of auxiliary activators when metabolism was reduced.

### Alternate models

The number of active sites firing transcription within a cell is limited by gene copy number, but the activated-DNA state in our simple linear model is unbounded. To test whether error frequency suppression persists when an upper bound on gene activity is introduced, we considered a simple two-state transcription model for which deterministic representation is given by:
(5)

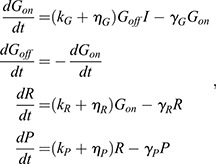


where *G_on_* and *G_off_* are the on- and off- states of a gene; *I*, *R* and *P* are the input, RNA and protein levels; and *k_i_*, γ*_i_* and η*_i_* are the primary synthesis, decay and auxiliary synthesis rate constants for species *i*, respectively. Rate parameter dependencies upon metabolic and protein synthesis conditions were analogous to those used in the linear model, and are shown in Table 3.

**Table 3.**
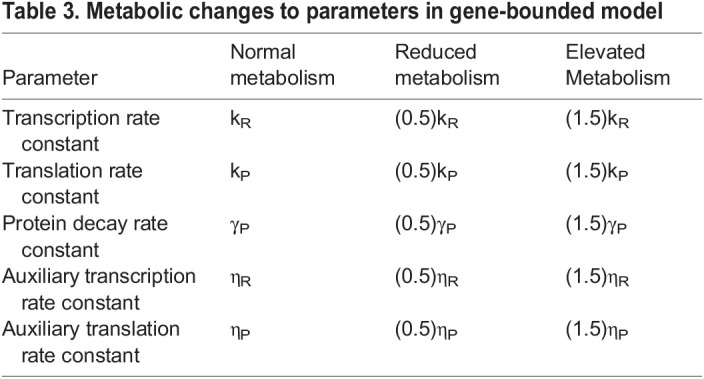
Metabolic changes to parameters in gene-bounded model

We performed a parameter sweep of this model in which all simulations were initialized as diploid (*G_off_*=2). Despite the limitation placed on gene activity, error frequency remains elevated under normal growth conditions and partially suppressed when metabolism is reduced (Fig. S2A).

Gene expression models also frequently utilize cooperative kinetics in order to capture the nonlinearities and thresholds encountered in transcriptional regulation. We reformulated our gene expression model in terms of Hill kinetics:
(6)

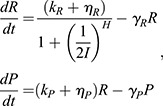
where *I*, *R* and *P* are the input, RNA and protein levels; *k_i_*, γ*_i_* and η*_i_* are the primary synthesis, decay and auxiliary synthesis rate constants for species *i*; and *H* is a transcriptional Hill coefficient. The stimulus level corresponding to half-maximal transcription rate was fixed at 0.5 because we only consider a binary input signal. Rate parameters were again scaled with metabolic conditions in a manner analogous to the linear model (Table 4).

**Table 4.**
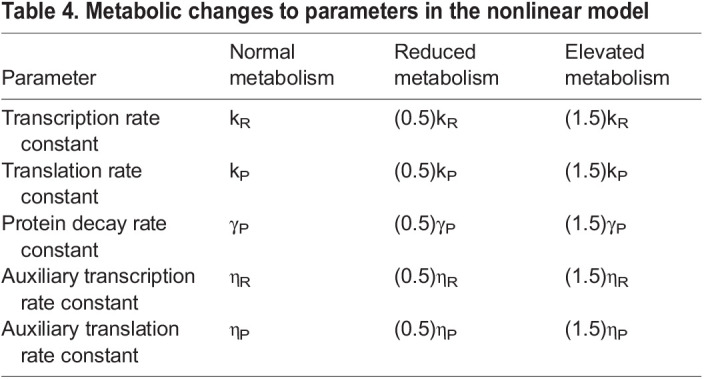
Metabolic changes to parameters in the nonlinear model

Another parameter sweep revealed that, despite the incorporation of cooperative binding kinetics, error frequency remains elevated under normal metabolic conditions and is broadly suppressed when metabolism is reduced (Fig. S2B).

### Quantification and statistics

Confidence intervals for the moving average of Yan-YFP expression were inferred from the 2.5th and 97.5th percentile of 1000 point estimates of the mean within each moving-average window. Point estimates were generated by bootstrap resampling with replacement of the expression levels within each window. Differences in dILP2 levels were tested by a Welch's *t*-test. Histogram distributions for the mean value of the *D* metric were calculated from 10,000 point estimates of the mean as generated by bootstrap resampling with replacement of *D* metric measures for each condition. Best-fit Gaussian distributions were fit onto each histogram. There was no exclusion of any data or subjects.

## Supplementary Material

10.1242/develop.201986_sup1 Supplementary information
