# Supplementary material for: Energy metabolism modulates the regulatory impact of activators on gene expression
Source: Development. 2024 Jan 2;151(1):dev201986. doi: 10.1242/dev.201986 (PMC10820824; doi:10.1242/dev.201986)
Supplement: Supplementary information [file develop-151-201986-s1.pdf]

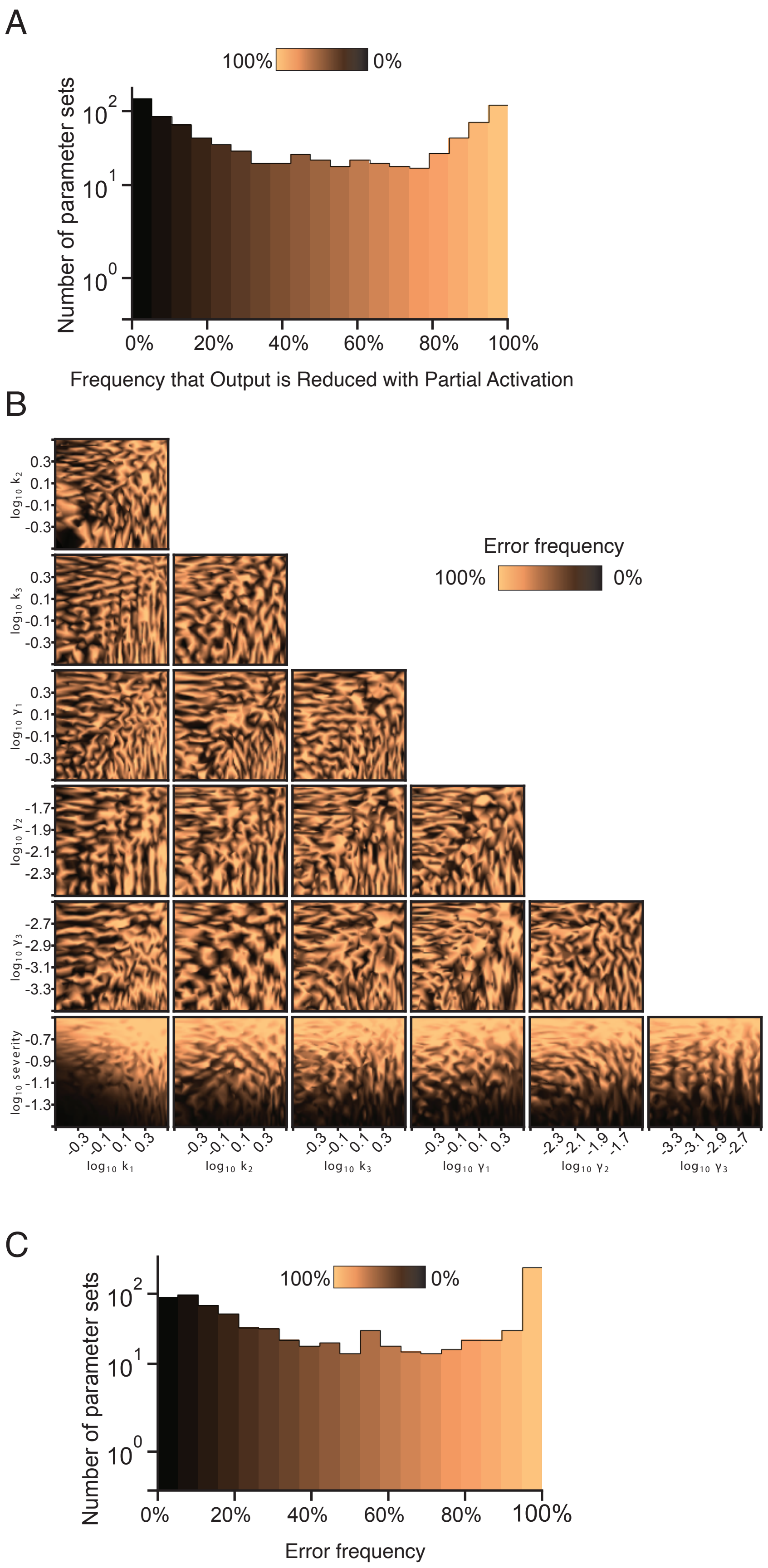

Fig. S1. Model expression dynamics are dependent on gene activators - related to Figure 1. Each of the seven model parameters was varied by one order of magnitude centered around the default value as defined in the Methods. 1,000 such variable parameter sets were generated. Simulations with full and partial activation were performed for each parameter set. **(A)** Protein output was compared between full and partial activation over the entire time course of gene expression. Shown is the frequency in which output reduction occurs with partial activation when compared to full activation for all parameter sets. **(B)** Error frequencies with partial activation were calculated using a threshold set at the lowest 1% of peak protein output from full activation. Shown is a grid of all 21 pairwise combinations of parameter variations. Error frequencies are projected as color heat maps on the 21 squares. Error frequencies are high (light brown) for many combinations of parameter values. **(C)** Distribution of error frequency for all parameter sets under conditions of normal energy metabolism.

Development • Supplementary information

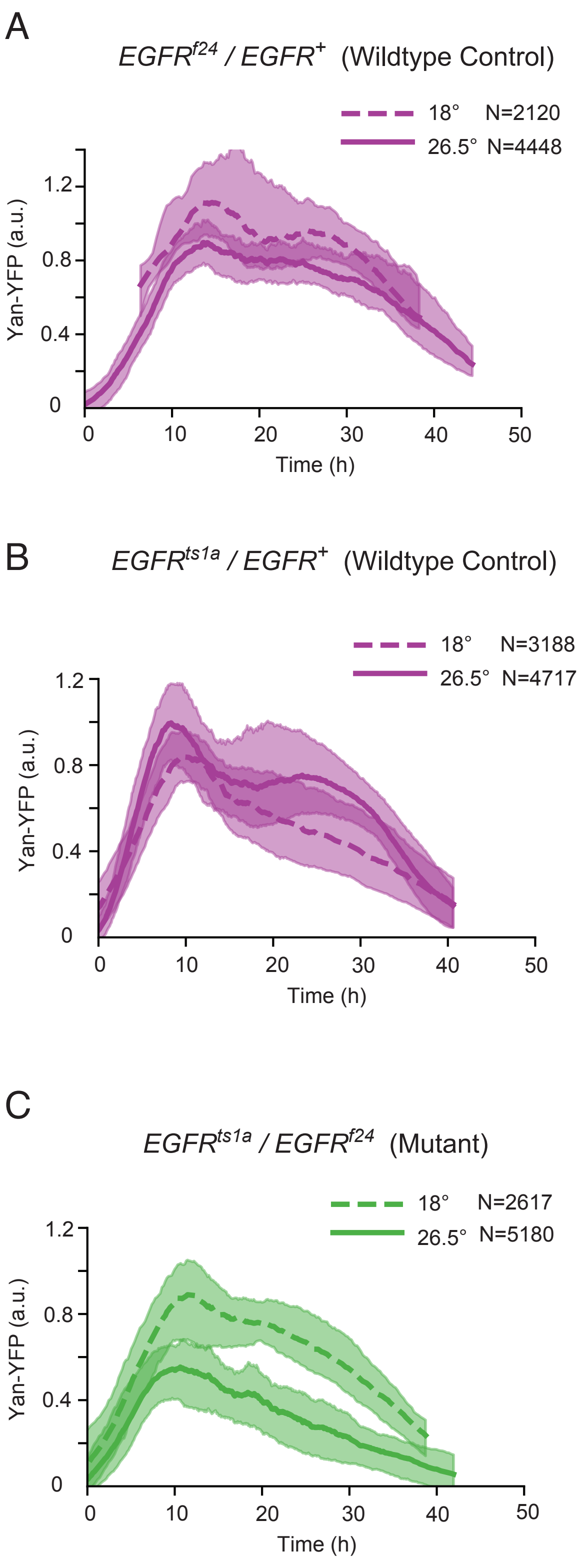

Fig. S2. Transient loss of EGFR activity causes lower Yan output - related to Figure 3. **(A)** Yan expression dynamics in *Egfr*^{f24}/+ heterozygous eyes after incubation in vivo at either 18°C or 26.5°C for 18 hours before fixation and analysis. N refers to number of single cells in which Yan measurements were made. **(B)** Yan expression dynamics in *Egfr*^{tsla}/+ heterozygous eyes after incubation in vivo at either 18°C or 26.5°C for 18 hours before fixation and analysis. N refers to number of single cells in which Yan measurements were made. **(C)** Yan expression dynamics in *Egfr*^{tsla}/*Egfr*^{f24} trans-heterozygous eyes after incubation in vivo at either 18°C or 26.5°C for 18 hours before fixation and analysis. N refers to number of single cells in which Yan measurements were made.

Development • Supplementary information

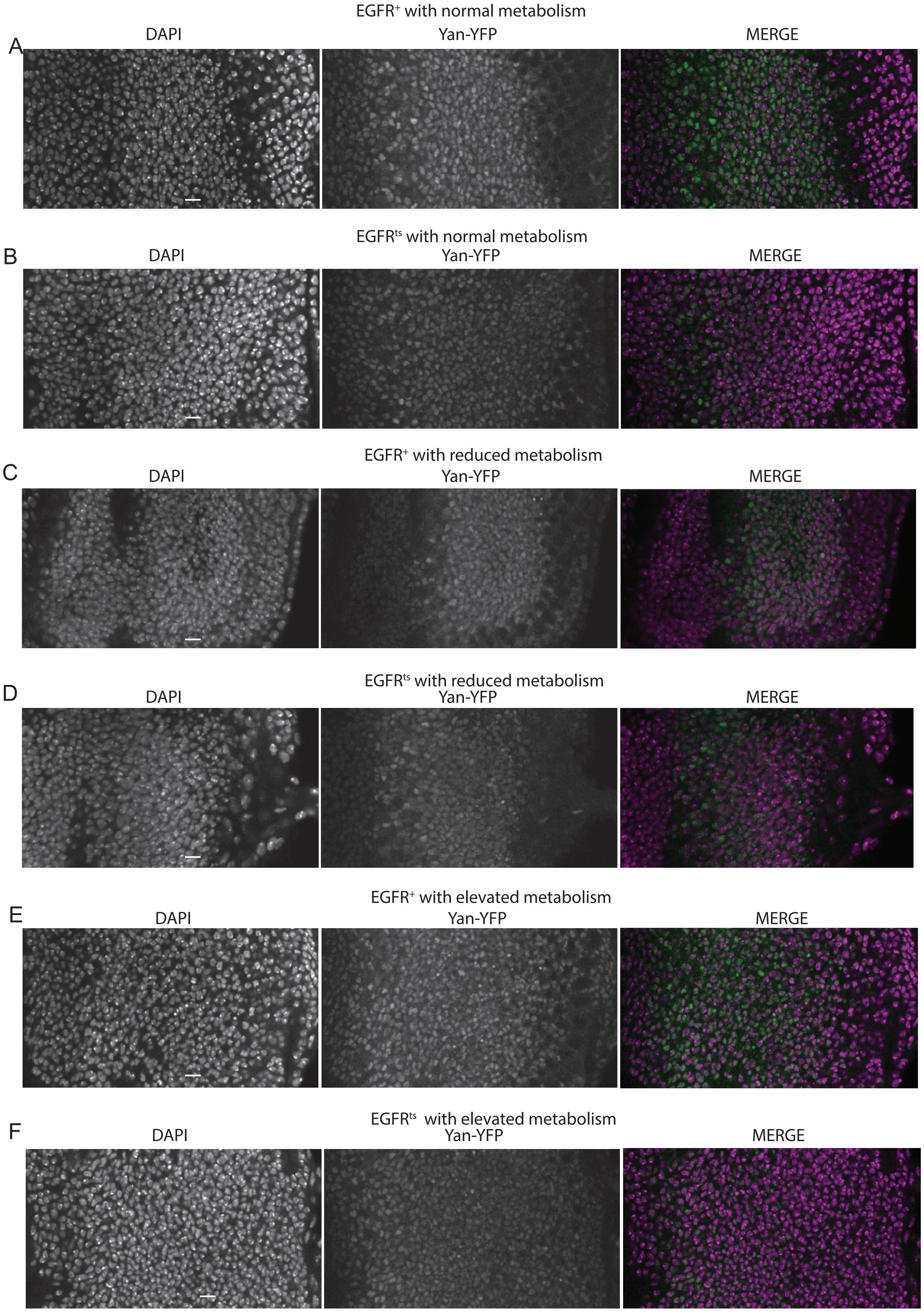

Fig. S3. Representative images of Yan expression in eye discs - related to Figure 3. All panels show a single optical slice through cell nuclei in the field of view taken of eye discs from animals that were incubated at 26.5°C for 18 hours. Left panels, DAPI channel; middle panels, Yan-YFP channel; right panels, merged channels with DAPI (purple) and Yan-YFP (green). Scale bars = 8 µm. **(A)** An *EGFR* wildtype animal with normal unperturbed metabolism. **(B)** An *EGFR* mutant animal with normal unperturbed metabolism. **(C)** An *EGFR* wildtype animal that is also *dILP2>Rpr*. **(D)** An *EGFR* mutant animal that is also *dILP2>Rpr*. **(E)** An *EGFR* wildtype animal that is also *GMR>Myc*. **(F)** An *EGFR* mutant animal that is also *GMR>Myc*.

Development • Supplementary information

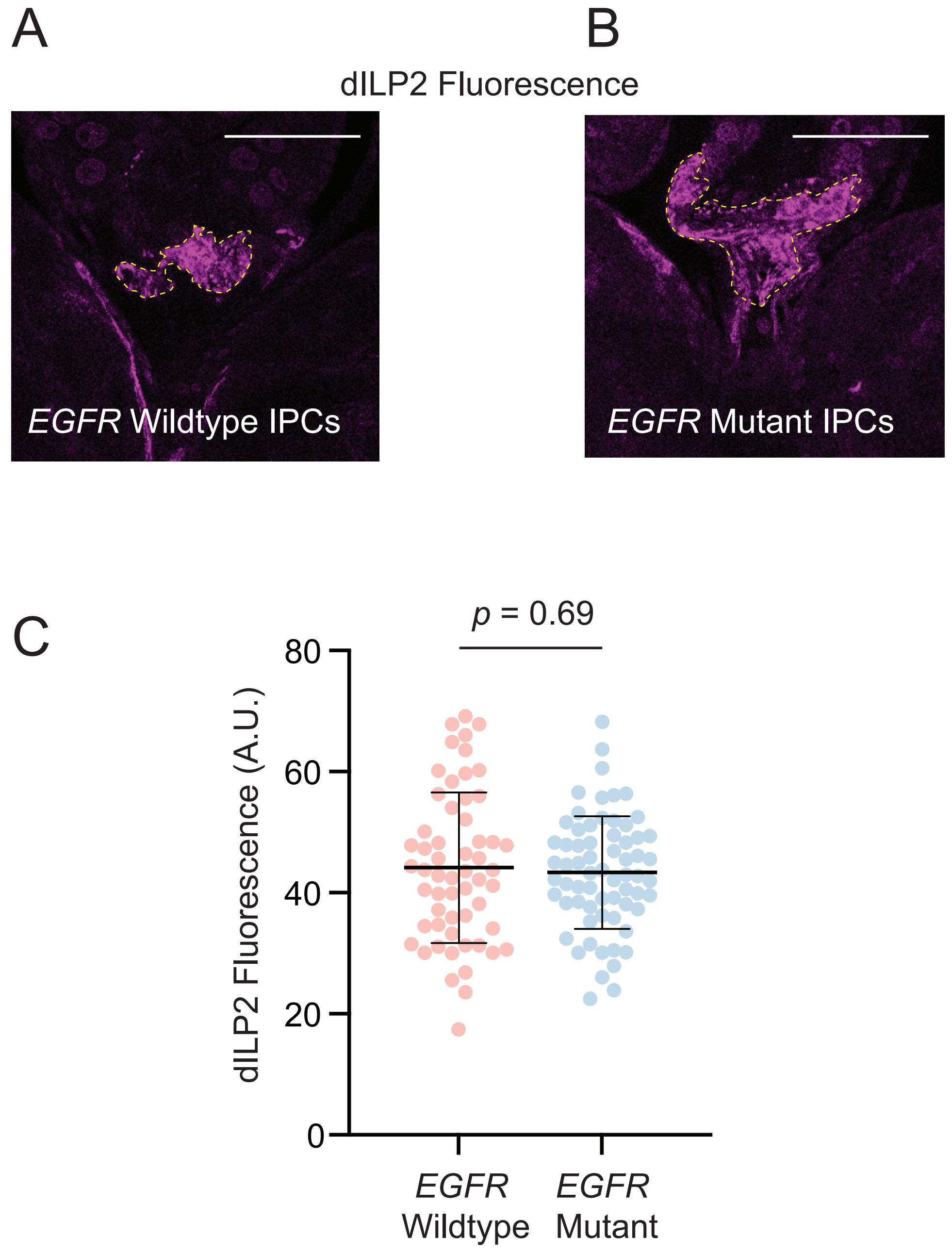

Fig. S4. dILP2 accumulation levels in IPCs - related to Figure 4. (A,B) dILP2 levels in the termini of IPCs, which are outlined. Animals had been incubated at 26.5°C for 16 hr prior to analysis in order to transiently inactivate the *EGFR*^{tsla} ts allele. Scale bars = 50 µm. **(A)** *EGFR*^{tsla} / + wildtype. **(B)** *EGFR*^{tsla} / *EGFR*^{f24} mutant. **(C)** Fluorescence intensity measurements of dILP2 in multiple IPC termini from larvae that were incubated at the semi-permissive temperature. Mean and standard deviations are shown. A Welch's t-test was performed to compare the two treatments.

Development • Supplementary information

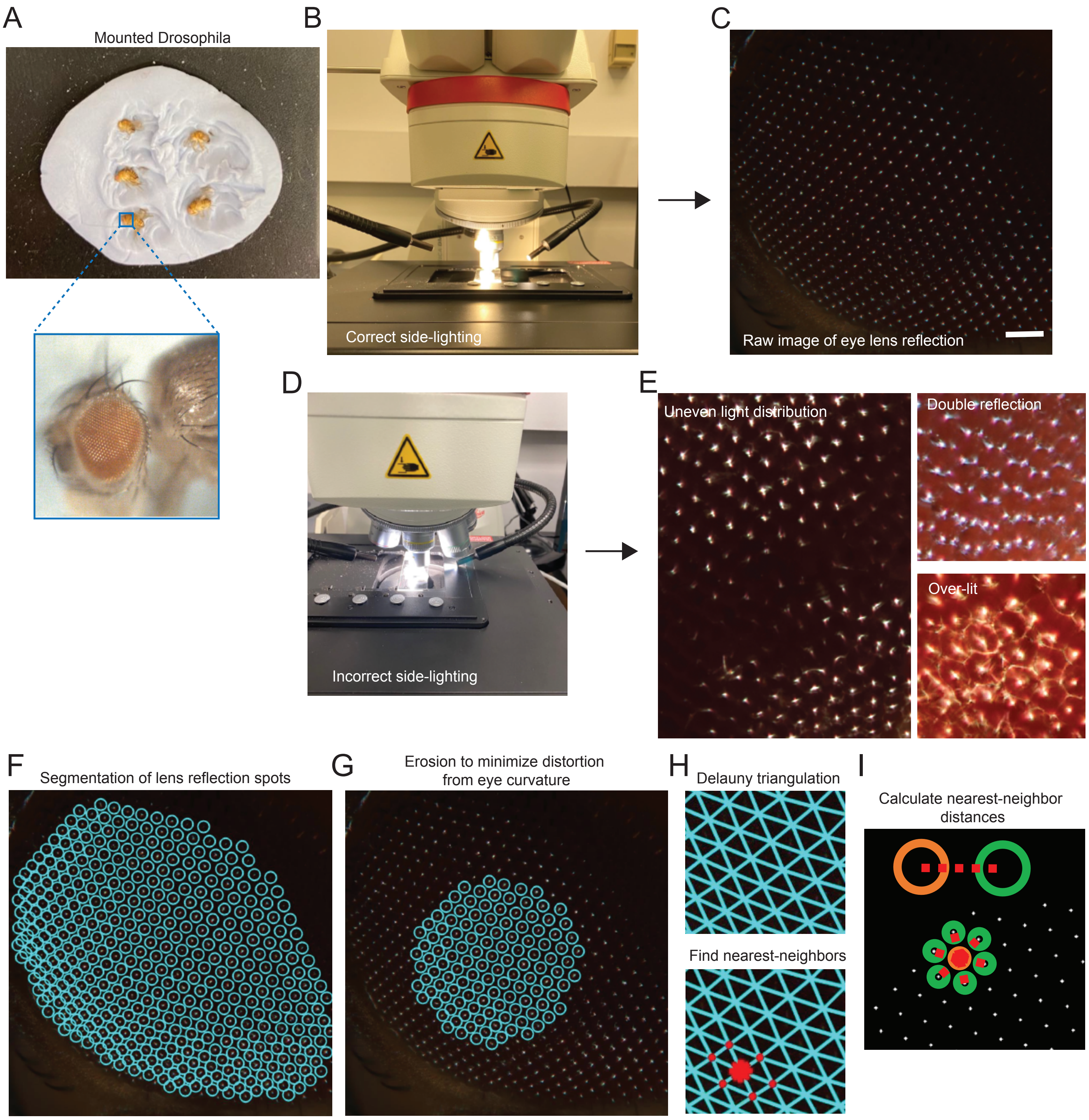

Fig. S5. Workflow for quantitative analysis of disorder in the compound eye - Related to Figure 5. (A) Sample adults mounted on Blu-Tack. **(B)** Correct configuration of fiber-optic lighting of samples on the Leica microscope stage. **(C)** Raw image of an eye using correct lighting and exposure conditions. Scale bar = 35μm. **(D)** Example of incorrect configuration of lighting in which extraneous objectives distort the lighting. **(E)** Resulting images with incorrect lighting and exposure are shown. **(F,G)** An image of an eye after segmentation of the ommatidia **(F)** and subsequent erosion of the segmented region of interest **(G)** to minimize distortion due to eye curvature. **(H)** Centroids within segmented ommatidia are interconnected by a Delaunay triangulation. The nearest neighbors of each centroid are identified. **(I)** Distances from each centroid to its nearest neighbors are calculated.

Development • Supplementary information

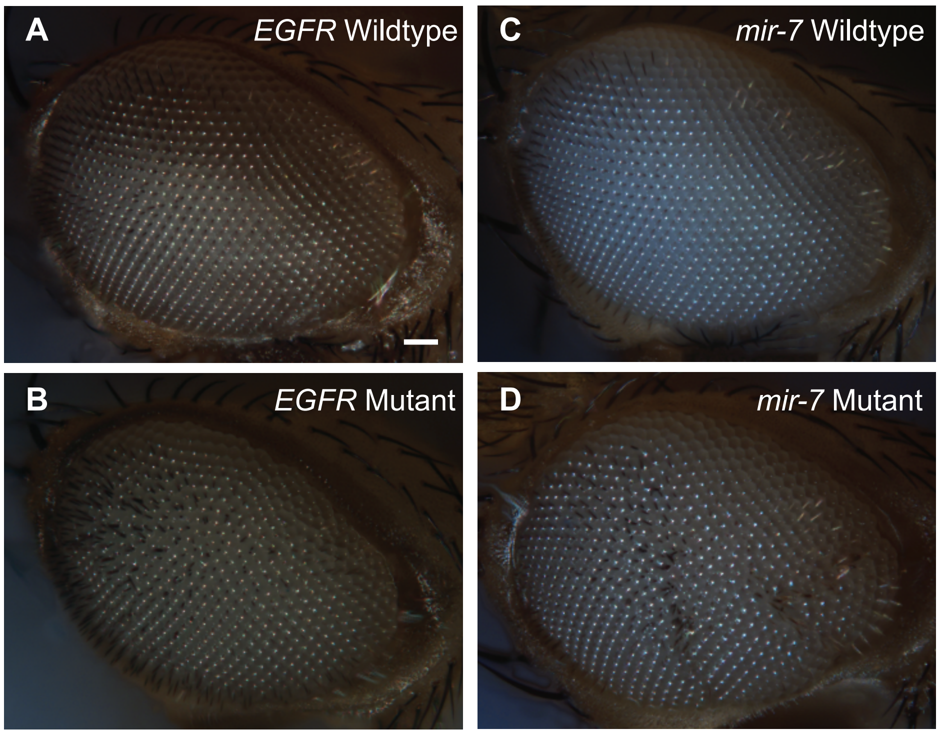

Fig. S6. Brightfield images of adult compound eyes - related to Figure 5. Shown are representative raw images of eyes for disorder quantitation. Lighting and exposure are adjusted to generate pointillistic reflection of light from ommatidial lenses. Scale bar = 35 μm. **(A)** $\textit{Egfr}^{f24}$ / + wildtype. **(B)** $\textit{Egfr}^{tsla}$ / $\textit{Egfr}^{f24}$ mutant. **(C)** $\textit{mir-7}^{\Delta 1}$ / + wildtype. **(D)** $\textit{mir-7}^{\Delta 1}$ mutant.

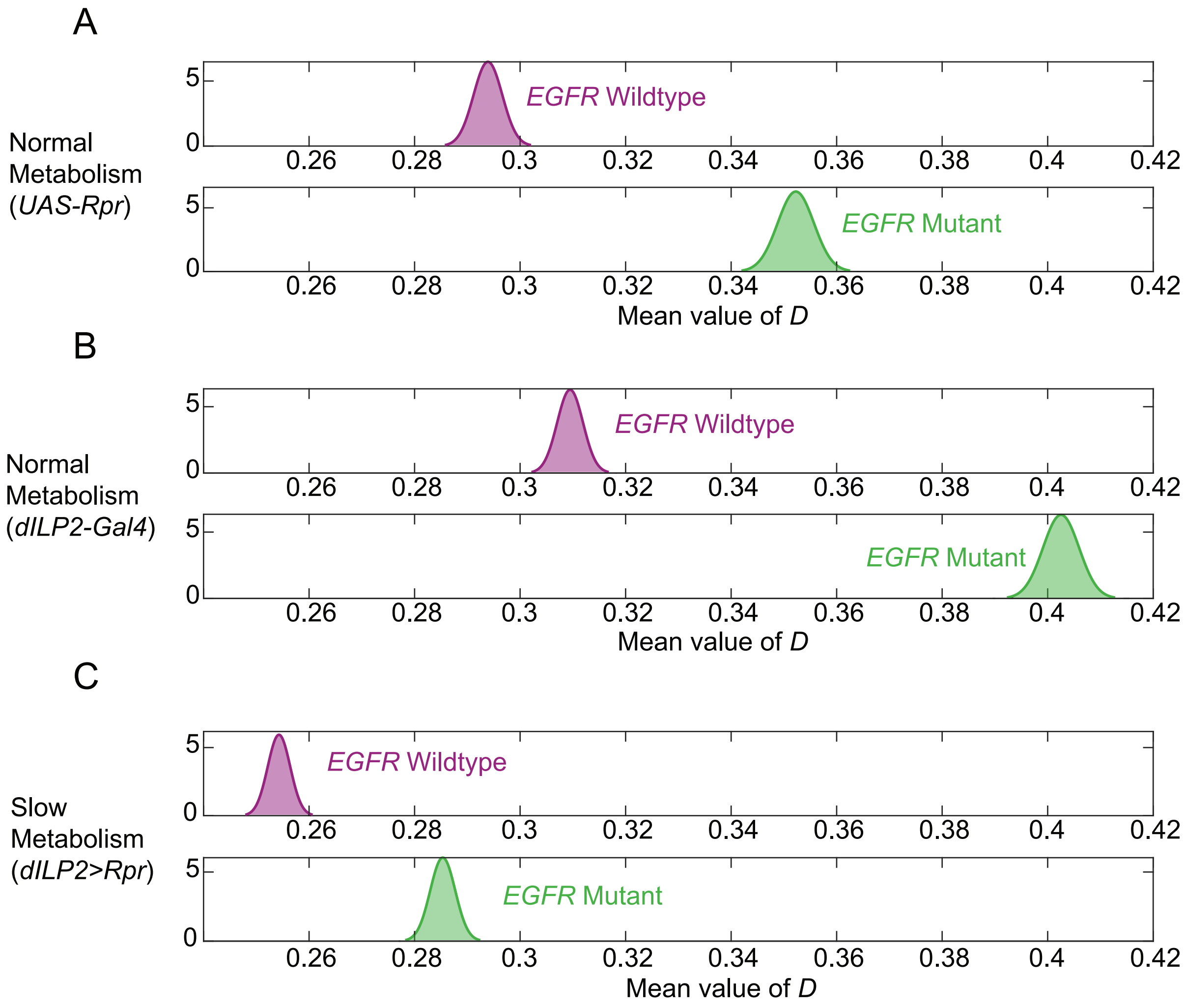

Fig. S7. Density distributions of the mean D for EGFR mutants with normal or reduced metabolism - related to Figure 6. Density distributions of the mean *D* were estimated for ommatidia from wildtype (purple) and *EGFR ts* mutant (green) eyes. All animals were raised at 18°C except for an 18-hour interval as late L3 larvae when they were incubated at 26.5°C. **(A,B)** Eye disorder in animals with normal metabolism. **(C)** Eye disorder in animals with reduced metabolism due to IPC ablation.
